# QTc measurement using Apple Watch electrocardiogram in congenital long QT syndrome

**DOI:** 10.1093/ehjdh/ztaf142

**Published:** 2026-01-12

**Authors:** Nicole J van Steijn, Auke T Bergeman, Christian van der Werf, Renee N A M Veenstra, Willem R van de Vijver, Ole G Helmink, G Aernout Somsen, Igor I Tulevski, Reinoud E Knops, Arthur A M Wilde, Michiel M Winter

**Affiliations:** Heart Center, Department of Clinical and Experimental Cardiology, Amsterdam UMC Location University of Amsterdam, Meibergdreef 9, AZ 1105 Amsterdam, The Netherlands; Amsterdam Cardiovascular Sciences, Heart Failure and Arrhythmias, Amsterdam, The Netherlands; Cardiology Centres of the Netherlands, Burgemeester Verderlaan 15, 3544 AD Utrecht, The Netherlands; Heart Center, Department of Clinical and Experimental Cardiology, Amsterdam UMC Location University of Amsterdam, Meibergdreef 9, AZ 1105 Amsterdam, The Netherlands; Amsterdam Cardiovascular Sciences, Heart Failure and Arrhythmias, Amsterdam, The Netherlands; Heart Center, Department of Clinical and Experimental Cardiology, Amsterdam UMC Location University of Amsterdam, Meibergdreef 9, AZ 1105 Amsterdam, The Netherlands; Amsterdam Cardiovascular Sciences, Heart Failure and Arrhythmias, Amsterdam, The Netherlands; Cardiology Centres of the Netherlands, Burgemeester Verderlaan 15, 3544 AD Utrecht, The Netherlands; Heart Center, Department of Clinical and Experimental Cardiology, Amsterdam UMC Location University of Amsterdam, Meibergdreef 9, AZ 1105 Amsterdam, The Netherlands; Heart Center, Department of Clinical and Experimental Cardiology, Amsterdam UMC Location University of Amsterdam, Meibergdreef 9, AZ 1105 Amsterdam, The Netherlands; Amsterdam Cardiovascular Sciences, Heart Failure and Arrhythmias, Amsterdam, The Netherlands; Cardiology Centres of the Netherlands, Burgemeester Verderlaan 15, 3544 AD Utrecht, The Netherlands; Department of Mechanical Engineering, Delft University of Technology, Mekelweg 5, 2628 CD Delft, The Netherlands; Cardiology Centres of the Netherlands, Burgemeester Verderlaan 15, 3544 AD Utrecht, The Netherlands; Cardiology Centres of the Netherlands, Burgemeester Verderlaan 15, 3544 AD Utrecht, The Netherlands; Heart Center, Department of Clinical and Experimental Cardiology, Amsterdam UMC Location University of Amsterdam, Meibergdreef 9, AZ 1105 Amsterdam, The Netherlands; Amsterdam Cardiovascular Sciences, Heart Failure and Arrhythmias, Amsterdam, The Netherlands; Heart Center, Department of Clinical and Experimental Cardiology, Amsterdam UMC Location University of Amsterdam, Meibergdreef 9, AZ 1105 Amsterdam, The Netherlands; Amsterdam Cardiovascular Sciences, Heart Failure and Arrhythmias, Amsterdam, The Netherlands; Cardiology Centres of the Netherlands, Burgemeester Verderlaan 15, 3544 AD Utrecht, The Netherlands; Heart Center, Department of Clinical and Experimental Cardiology, Amsterdam UMC Location University of Amsterdam, Meibergdreef 9, AZ 1105 Amsterdam, The Netherlands; Amsterdam Cardiovascular Sciences, Heart Failure and Arrhythmias, Amsterdam, The Netherlands; Cardiology Centres of the Netherlands, Burgemeester Verderlaan 15, 3544 AD Utrecht, The Netherlands

**Keywords:** Mobile health, Wearable technology, Single-lead ECG, Long QT syndrome

## Abstract

**Aims:**

In congenital long QT syndrome (cLQTS), monitoring of the heart rate–corrected QT interval (QTc) is essential as even transient prolongation can significantly increase the risk of torsades de pointes and sudden cardiac death. Apple Watch (AW) offers a single-lead mobile electrocardiogram (mECG), but its accuracy for QTc monitoring remains uncertain. The objective is to analytically validate AW mECGs for QTc measurement in paediatric and adult cLQTS patients, assessing agreement, systematic bias, and lead-specific feasibility compared with standard 12-lead ECG.

**Methods and results:**

In this cross-sectional, dual-centre study, patients with cLQTS underwent consecutive 12-lead ECG, followed by mECG recordings of Leads I and II. QT intervals were measured by two blinded investigators, and accuracy was evaluated using Bland–Altman analysis. The study was deemed exempt from formal ethical approval by the Medical Ethics Committee of the Amsterdam UMC. Of 101 patients enrolled, 99 had ECGs suitable for QTc analysis; 15 (15.2%) were younger than 18 years and 62 (62.6%) were female. On 12-lead ECG, the mean QTc was 444.9 ± 30.2 ms (Lead I) and 449.0 ± 29.8 ms (Lead II), compared with 466.6 ± 28.9 ms (Lead I) and 470.0 ± 29.8 ms (Lead II) on mECG. The mean QTc difference (12-lead—AW) was −21.7 ms (95% limits of agreement: −53.1–9.7) for Lead I and −21.0 ms (−59.5–17.5) for Lead II.

**Conclusion:**

In patients with cLQTS, AW-derived mECGs may complement, but not replace, standard 12-lead ECGs for QTc assessment, pending further validation in longitudinal and unsupervised settings.

## Graphical Abstract

**Graphical Abstract:**
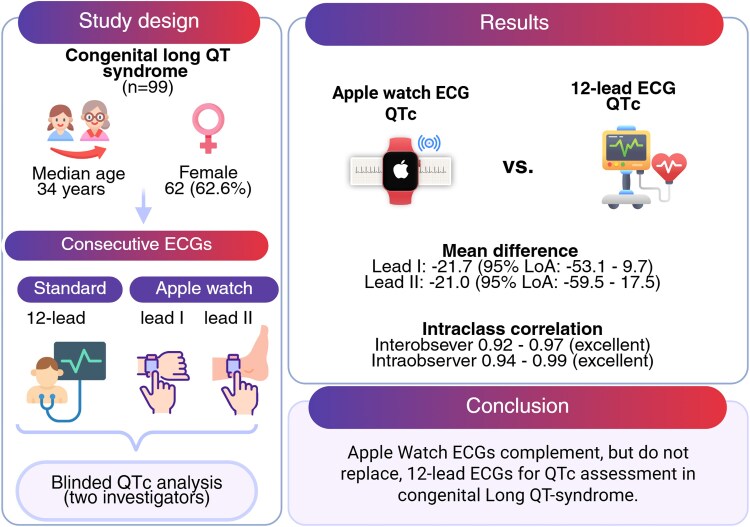
Accuracy of Apple Watch single-lead ECG for QTc assessment in congenital long QT syndrome. Study overview showing single-lead Apple Watch mECG and 12-lead ECG QTc measurements for both Leads I and II in patients with congenital long QT syndrome. Mean difference was modest, but wide limits of agreement limit suitability for unsupervised clinical use.

## Introduction

In congenital long QT syndrome (cLQTS), monitoring of the heart rate–corrected QT interval (QTc) is an important component of risk assessment, as even transient prolongation can predispose to torsades de pointes.^1,2^ Standard QTc evaluation relies on a 12-lead electrocardiogram (ECG) which requires in-office acquisition and provides only brief snapshots of cardiac repolarisation. This limits the ability to capture dynamic QTc changes that may occur during daily life or in response to genotype-specific triggers such as exercise or emotional stress.^3^

Wearable single-lead mobile ECGs (mECGs) could facilitate ECG recording outside the clinical setting and thereby enable on-demand QTc assessment. However, before such applications can be considered, the basic feasibility and accuracy of QTc measurement from these devices must be established. Among available wearables, the Apple Watch [Apple Inc., Cupertino, CA, USA (AW)] is the most widely used consumer device with integrated single-lead ECG functionality and thus offers a scalable platform for remote monitoring.

However, evidence on the validity of AW-derived QTc measurements in cLQTS remains scarce. In this population, QTc assessment is particularly challenging because genotype-specific T-wave morphologies can hinder consistent delineation of the QT interval.^4,5^ Preliminary reports have explored non-standard configurations, such as placing the watch on the chest (approximating Lead V5) or along a Lead II vector,^6^ but the reliability of the standard wrist-based (Lead I) configuration, which underlies most real-world recordings, has not been systematically evaluated.

Therefore, this study aims to analytically validate AW single-lead mECGs for QTc measurement in paediatric and adult patients with cLQTS, quantifying agreement with the reference 12-lead ECG, systematic bias, and lead-specific feasibility.

## Methods

### Study design and participants

The Medical Ethics Review Committee of the Amsterdam University Medical Center reviewed the study and determined that it was not subject to the Medical Research Involving Human Subjects Act (WMO) (reference number: W22_384 # 22.491). The study was conducted in accordance with the principles of the Declaration of Helsinki.

In this dual-centre, cross-sectional validation study, we included both paediatric and adult patients with cLQTS who had a scheduled clinic visit between 5 December 2022 and 17 March 2025 at the Amsterdam University Medical Center (Amsterdam, The Netherlands) or at Cardiology Centers of the Netherlands (Amsterdam, The Netherlands). Written informed consent was obtained from all patients or from their legal representative in case of patients under the age of 16. Patients were excluded if their ECGs displayed atrial fibrillation or a complete bundle branch block. Moreover, we excluded patients with a pacemaker whose ECGs did not display five consecutive non-ventricularly paced QRS complexes.

### Study procedures

**Figure 1:**
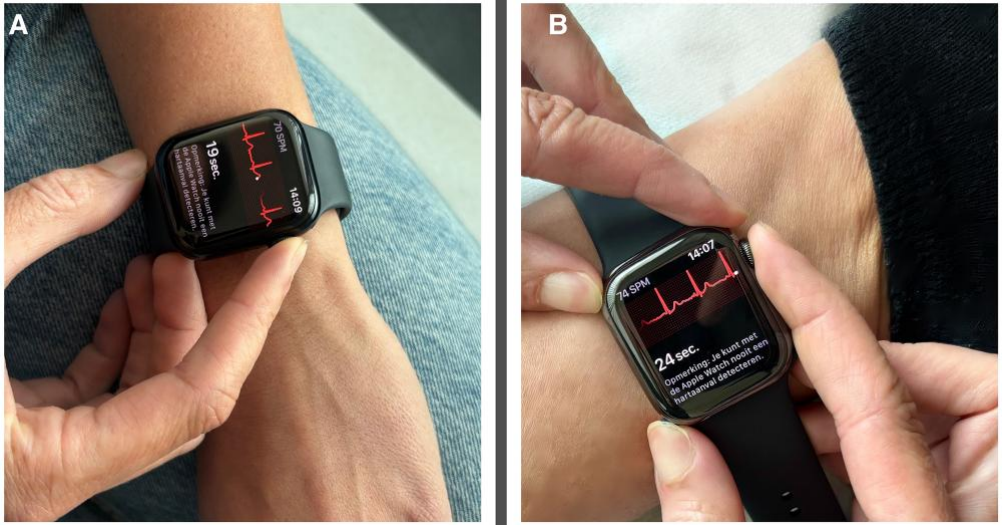
Creating a single-lead mobile electrocardiogram using an Apple Watch. Apple Watch contains one electrode on the back of the device and one on the side-mounted dial. A 30-s single-lead electrocardiogram can be obtained in different lead configurations: (*A*) the watch is worn on the left wrist, and the right index finger touches the side-mounted dial to record Lead I (the conventional and most used configuration). (*B*) The watch is worn on the left ankle, and the right index finger touches the digital crown to approximate Lead II. This figure illustrates the versatility of smartwatch-based electrocardiogram acquisition for remote rhythm monitoring. ECG, electrocardiogram.

**Figure 2:**
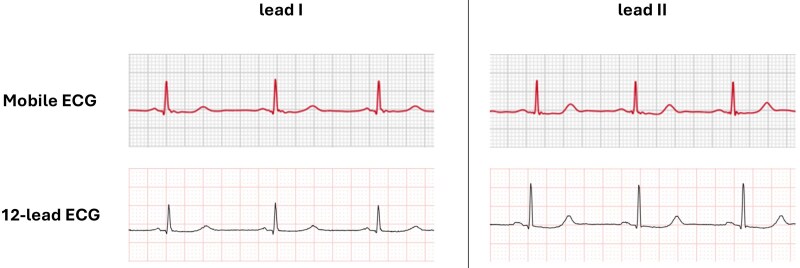
Example of mobile electrocardiogram and 12-lead electrocardiogram in a patient with congenital long QT syndrome. This figure illustrates a comparison of QTc intervals recorded in a patient with congenital long QT syndrome using a single-lead mobile electrocardiogram (smartwatch) and a standard 12-lead electrocardiogram. The upper panels show segments of 30-s mobile electrocardiogram tracings acquired in Lead I (upper left) and Lead II (upper right) configurations. The lower panels display the corresponding leads from 12-lead electrocardiograms. QT intervals were manually measured using the tangent method and were corrected for heart rate using Bazett’s formula. Mobile electrocardiogram tracings show an overestimation of QTc intervals compared with 12-lead electrocardiogram: 504.9 vs. 485.3, for Lead I, and 486.4 vs. 483.1 for Lead II. ECG, electrocardiogram; QTc, corrected QT interval; mECG, mobile ECG.

First, a 12-lead ECG was recorded with the patient in a semi-recumbent Fowler’s position (≈45°), to enable subsequent mECG acquisition with comparable posture, limiting heart rate (HR) fluctuations. Immediately thereafter, two AW Series 8 mECGs were obtained. The AW contains two electrodes: on the back of the watch and one on the side-mounted control dial. A 30-s single-lead mECG is created by touching the control dial with the right index finger and placing the back of the watch on the left wrist (for Lead I) or left ankle (for Lead II). QTc is preferably measured in Lead II because of its alignment with the cardiac electrical axis and typical clarity of the T-wave, although Lead I can provide a suitable alternative when T-waves are not well visualised. We included both Lead I and Lead II to reflect real-world use and to evaluate lead-specific feasibility, as AW records Lead I by default.^7^ Examples of smartwatch positions and ECG tracings are shown in *Figures 1* and *2*, respectively.

### Annotation procedures

All ECGs were acquired using the manufacturer default diagnostic settings, exported in digital format, and deidentified prior to analysis. Three consecutive QRS complexes were marked for measurement by an independent research fellow; if artefacts precluded measurement of three complexes, two were selected. Measurements were performed using EP Calipers software (EP Studios, Inc., Louisville, KY, USA). For each ECG, two blinded researchers (A.T.B. and N.J.v.S.) manually measured QT intervals and the corresponding preceding RR intervals in the marked complexes. The end of the T-wave was determined using the tangent method,^7^ and QTc was calculated using Bazett’s formula (QT/√RR). To avoid bias from consensus discussions, both investigators performed measurements independently, and the mean of their QTc values was used for analysis. Interobserver and intraobserver reliability were assessed using intraclass correlation coefficients (ICCs) based on repeated measurements of a random subset of ECGs.

### Statistical analysis

Categorical variables are presented as frequencies and percentages. Continuous variables are presented as median with interquartile range (IQR) for consistency across variables. Heart rate was compared between devices using the Wilcoxon signed-rank test. Mean differences (QTc_12−lead_ − QTc_mECG_) were calculated for each paired measurement. A negative mean difference indicates the overestimation of the QTc by mECG. Bland–Altman analysis was used to quantify bias and limits of agreement (LOA), and a paired Student’s *t*-test was performed to assess whether the mean difference differed significantly from zero. The 95% LOA was defined as the mean difference ±1.96 × the standard deviation of the differences, representing the range within which 95% of differences between methods are expected to lie.^8,9^ Perfect agreement was defined as an absolute difference of <10 ms, and acceptable agreement as a difference of 10–20 ms.

Interobserver reliability was assessed using a two-way mixed-effects model for consistency, with subjects considered a random effect and the two observers a fixed effect, as the same observers performed all measurements. Intraobserver reliability was assessed using a two-way mixed-effects model for *absolute agreement*. ICC values were interpreted as follows: <0.50, poor agreement; 0.50–0.75, moderate agreement; 0.75–0.90, good agreement; and >0.90, excellent agreement.^10^ For intraobserver assessment, an independent research fellow randomly selected 10% of all deidentified mECG and 12-lead ECG recordings, which were reassigned newly deidentified numbers before being remeasured. Electrocardiogram recordings that were qualitatively insufficient for at least two QTc measurements (e.g. due to excessive noise, artefacts, or flat T-waves) were excluded.

Statistical analysis was performed using R, version 4.3.2 (R Foundation for Statistical Computing, Vienna, Austria), in RStudio, version 2023.12.1 (Posit, PBC, Boston, MA, USA). A two-tailed probability value of <0.05 was considered statistically significant.

## Results

### Study population

We enrolled 101 patients with cLQTS in the study. Fifteen (15.2%) patients were younger than 18 years and 62 (62.6%) were female (*Table 1*). All patients carried a pathogenic variant in an LQTS-associated gene, most commonly in *KCNH2* (41 patients, 41.4%), followed by *KCNQ1* (34 patients, 34.3%), *SCN5A* (20 patients, 20.2%), *CACNA1C* (3 patients, 3.0%), and *KCNE1* (1 patient, 1.0%).

**Table 1:** Demographic and clinical characteristics of the patients at baseline

Characteristic	All patients (*n* = 99)
Age—years, median (IQR)	34 (22–49)
Paediatric patients—no. (%)	15 (15.2)
Female sex—no. (%)	62 (62.6)
BMI—kg/m^2^, median (IQR)	23.5 (20.7–26.2)
Management—no. (%)	
Beta-blockers	63 (63.6)
Mexiletine	4 (4.0)
Pacemaker	6 (6.1)
Dual-chamber pacing	4/6
Single-chamber pacing	2/6
Implantable cardioverter defibrillator	14 (14.1)
Primary prevention ICD	9/14
Secondary prevention ICD	5/14

BMI, body mass index; CMP, cardiomyopathy; IQR, interquartile range; SD; standard deviation.

### Electrocardiogram data quality

The median time interval between the 12-lead ECG and the mECG recording was 1.0 min (IQR 0.5–2.0) for Lead I and 3.5 min (IQR 2.5–5.0) for Lead II. In total, 95 (94.1%) patients were in sinus rhythm, and 6 (5.9%) had an atrially paced rhythm. In most cases, the quality of the ECGs allowed for QTc measurements in three consecutive complexes (12-lead ECG Lead I: 100%; 12-lead ECG Lead II: 96.1%; AW Lead I: 94.1%; AW Lead II 90.2%). Ninety-nine patients had at least one recording (Lead I or II) of sufficient quality for analysis of both the mECG and the corresponding 12-lead ECG lead. For Lead I, three patients were excluded from analysis due to flat T-waves (*n* = 2) or artefacts (*n* = 1) on the mECG. For Lead II, 8 patients were excluded from analysis due to flat T-waves (*n* = 5) or artefacts (*n* = 3) on either 12-lead or mECG, or both (see Supplementary material online, *Table S1*). Exclusion due to flat T-waves only occurred in carriers of *KCNH2* (*n* = 6) and *SCN5A* (*n* = 2) variants.

### Agreement between devices

The mean QTc measured on Lead I (*n* = 98) was 444.9 ± 30.2 ms on the 12-lead ECG and 466.6 ± 28.9 ms on the mECG. For Lead II (*n* = 93), the mean QTc duration was 449.0 ± 29.8 ms on the 12-lead ECG and 470.0 ± 29.8 ms on mECG. Median HRs for Lead I were 60 b.p.m. (IQR 53–69) and 62 b.p.m. (IQR 54–70) on 12-lead ECG and mECG, respectively (*P* = 0.12). For Lead II, the median HRs were 60 b.p.m. (IQR 53–69) and 65 b.p.m. (IQR 59–71) on 12-lead ECG and mECG (*P* < 0.0001).

**Figure 3:**
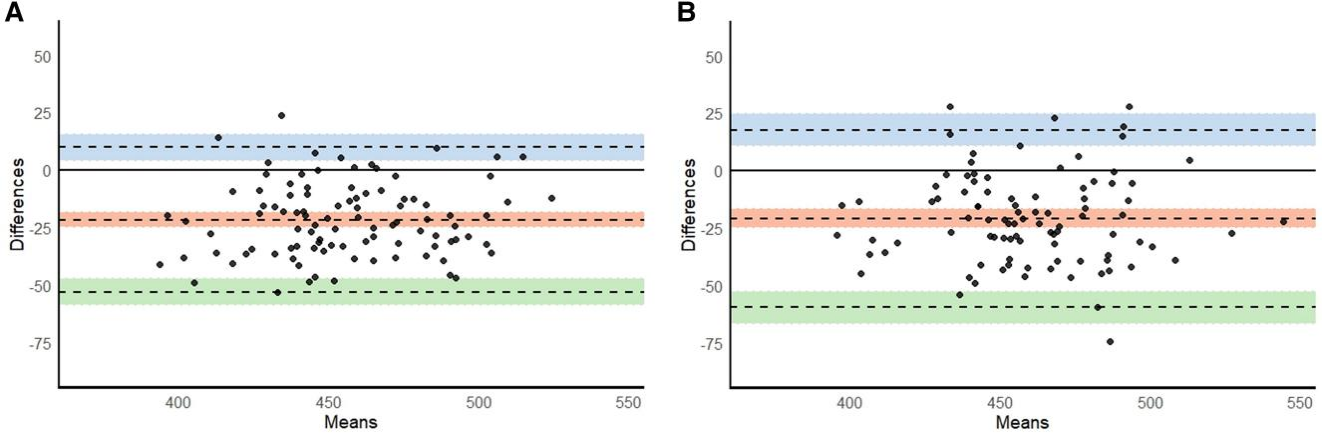
Agreement between mobile electrocardiogram and 12-lead electrocardiogram QTc measurements in two lead configurations. (*A*) Bland–Altman plot comparing QTc intervals from Lead I on single-lead mobile electrocardiogram and 12-lead electrocardiogram. (*B*) Bland–Altman plot comparing QTc intervals from Lead II on single-lead mobile electrocardiogram and 12-lead electrocardiogram. Each plot shows the mean QTc (*x*-axis) against the difference between single-lead and 12-lead QTc values (*y*-axis). The middle dashed line represents the mean difference, with its 95% confidence interval. The upper and lower dashed lines represent the limits of agreement, each shown with their corresponding 95% confidence intervals. Mean difference was defined as QTc_12−lead_ − QTc_mECG_. A negative mean difference indicates the overestimation of the QTc by mobile electrocardiogram. CI, confidence interval; ECG, electrocardiogram; mECG, mobile ECG.

The mean difference in QTc between mECG and 12-lead ECG Lead I was −21.7 ms (95% LOA −53.1 to 9.7, *P* < 0.0001). The mean difference in QTc between mECG and 12-lead ECG Lead II was −21.0 ms (95% LOA −59.5 to 17.5, *P* < 0.0001) (*Figure 3*). Among all genotype-lead combinations analysed, the QTc difference in *SCN5A* patients using Lead II showed the smallest difference (mean difference: 16.6 ms; 95% LOA: −67.5 to 34.2). Detailed results for all genotype-lead combinations are provided in Supplementary material online, *Table S2*.

The proportion of measurements showing perfect agreement was 20% in Lead I and 19% in Lead II. The proportion of measurements showing acceptable agreement was 45% for Lead I and 42% for Lead II. Moreover, for Lead I, 18, 16, and 10% of measurements deviated by 20–30, 30–40, and ≥40 ms, respectively; for Lead II, the corresponding proportions were 26, 15, and 17%. Supplementary material online, *Figure S1* shows the distribution of QTc agreement categories by genetic variant.

### Interobserver and intraobserver reliability

The interobserver reliability was 0.97 [95% confidence interval (CI): 0.75–0.99] and 0.96 (95% CI: 0.84–0.98) for 12-lead ECG Lead I and Lead II, respectively, and 0.94 (95% CI: 0.78–0.97) and 0.92 (95% CI: 0.85–0.95) for mECG Lead I and Lead II.

The intraobserver reliability demonstrated excellent reproducibility of QTc measurements upon repeated assessment. For the first researcher, ICCs were 0.98 (95% CI: 0.92–1.0, *P* < 0.0001) and 0.96 (95% CI: 0.86–1.0, *P* < 0.0001) for 12-lead ECG Leads I and II, respectively, and 0.99 (95% CI: 0.94–0.97, *P* < 0.0001) and 0.99 (95% CI: 0.95–1.0, *P* < 0.0001) for mECG Leads I and II. For the second researcher, ICCs were 0.96 (95% CI 0.84–0.99, *P* < 0.0001) and 0.97 (95% CI: 0.84–0.99, *P* < 0.0001) for 12-lead ECG Lead I and Lead II, respectively, and 0.99 (95% CI: 0.97–1.0, *P* < 0.0001) and 0.94 (95% CI: 0.57–0.99, *P* = 0.0025) for mECG Leads I and II.

## Discussion

In this cross-sectional study, we assessed the accuracy of QTc measurements derived from AW single-lead mECGs in 101 patients with congenital long QT syndrome. In both Lead I and Lead II configurations, AW mECG-derived QTc intervals differed from the reference 12-lead ECG by an average of 21 ms. Approximately 45% of measurements fell within our predefined threshold for acceptable agreement. Inter- and intraobserver reliability was excellent. Lead I enabled QTc measurement in 80% of cases in which Lead II was uninterpretable.

Multiple factors may explain the overestimation of mECG-derived QTc intervals. First, HR during mECG recordings was modestly higher than during resting 12-lead ECGs, particularly in Lead II, possibly due to body position and device handling. As Bazett’s correction tends to overestimate QTc at higher HRs,^11^ this difference in HR could have contributed to the longer QTc values observed on mECG. In addition, minor beat-to-beat or temporal variability in QT duration can occur even at stable HRs, although recordings were obtained consecutively, minimising this source of variation. However, the degree of bias observed across both leads suggests that HR and temporal variability alone do not account for the observed QTc overestimation. Morphology-related factors likely contributed as well. Characteristic T-wave abnormalities in cLQTS, such as low amplitude, notching, or prolonged repolarisation, can complicate end-T delineation and thereby affect QT measurement accuracy. This effect may be most pronounced in *KCNH2* carriers, who typically exhibit low-amplitude or bifid T-waves.^12^ Minor technical differences between mECG and standard 12-lead ECG systems, such as sampling frequency, signal filtering, and automatic amplitude scaling for display, may introduce subtle variation in waveform morphology and QTc measurement, which is inherent to cross-platform comparisons.

Our findings are consistent with previous literature showing that AW mECG-derived QTc intervals tend to overestimate QTc duration. Multiple studies demonstrated strong correlations and reported mean differences between 12-lead ECG and mECG QTc of <20 ms in patients with and without LQTS.^6,13,14^ However, the wide LOA observed across studies indicate that, while AW mECG-derived QTc may capture average differences across groups or conditions reasonably well, their accuracy for individual assessment remains limited. The slightly greater overestimation observed in our study is consistent with the study by Yee-Ming Li *et al*.^14^ showing poorer agreement in LQTS patients compared with those without QT abnormalities. A study in healthy volunteers reported only moderate correlation for QTc despite good agreement for raw QT intervals, underscoring the additional variability introduced by HR correction formulas.^15^

Our study advances prior studies by systematically evaluating both Lead I, the device’s default configuration, and Lead II, the two most practical configurations for home recording, and by enrolling genetically confirmed cLQTS patients. This design allowed us to examine device performance in a population characterised by complex T-wave morphology, who are most likely to benefit from enhanced QTc monitoring. We also applied more stringent criteria for defining agreement than most previous studies, which typically regarded differences of <20 and <40 ms as strong and moderate agreement, respectively.^15,16^ Given that even small QTc deviations can influence risk stratification and therapy in congenital LQTS, we defined <10 ms as perfect and <20 ms as acceptable agreement, omitting a broader ‘moderate’ category.

Before implementation in clinical practice, several factors should be considered. It is crucial to ensure patient safety and mitigate the risk of misinterpretation and patient anxiety whilst avoiding unnecessary investigations or treatment changes due to systematic overestimation. Moreover, although validated automated QTc algorithms have been developed for a consumer handheld mECG device,^17^ manual QTc adjudication by trained experts remains the recommended approach in cLQTS, even on standard 12-lead ECG.^18^ In practice, QTc monitoring would focus on relative changes within the same patient and device. As our study included only single measurements, within-patient comparisons should be interpreted with caution until supported by longitudinal data.

To facilitate safe and reliable implementation of smartwatch-based QTc monitoring in clinical practice, we propose the following pragmatic approach: (i) obtain two supervised, repeated measurements of baseline AW mECG and 12-lead ECGs to characterise individual bias, confirm signal quality, and decide individual suitability for remote QTc monitoring; (ii) advise patients to record ECGs at rest and consistently use the same lead configuration; and (iii) QTc measurements should be performed manually, by trained experts.

Certain limitations of this study need to be considered when extrapolating our findings to routine home monitoring. This study was performed in a controlled setting with supervised ECG acquisition and may not reflect real-world use. While a baseline comparison with a 12-lead ECG can identify patient-specific systematic bias, it does not reduce the intrinsic variability reflected by the wide LOA; therefore, AW mECG QTc values are not suitable for unsupervised decision-making in cLQTS. A number of mECGs were excluded because of insufficient signal quality or flat T-waves, particularly in *KCNH2* carriers, underscoring the technical and morphological challenges of remote QTc assessment. In addition, sequential rather than simultaneous recordings may have introduced minor physiological variability, although acquisition was immediate to minimise differences.

## Conclusions

In cLQTS, AW-derived mECGs could complement but not replace conventional 12-lead ECGs, provided that individual suitability for remote QTc monitoring has been assessed under supervised conditions. Future studies should evaluate performance in longitudinal and unsupervised real-world settings.

## Lead author biography


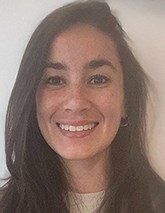


Nicole J. van Steijn is a PhD fellow and cardiology resident at Amsterdam University Medical Center. Her research focuses on wearable technology for detecting cardiac arrhythmias, with an emphasis on atrial fibrillation screening, early cardiac arrest detection, and QT interval assessment using single-lead ECG devices. She is particularly interested in the integration of digital health and telemonitoring in high-risk cardiovascular patient populations.

## Supplementary Material

ztaf142_Supplementary_Data

## Data Availability

The data underlying this article will be shared on reasonable request to the corresponding author.
