# Supplementary material for: QTc measurement using Apple Watch electrocardiogram in congenital long QT syndrome
Source: Eur Heart J Digit Health. 2026 Jan 12;7(3):ztaf142. doi: 10.1093/ehjdh/ztaf142 (PMC12994466; doi:10.1093/ehjdh/ztaf142)
Supplement: ztaf142_Supplementary_Data [file ztaf142_supplementary_data.docx]

**QTc Measurement using Apple Watch electrocardiogram in**

**congenital long QT syndrome**

Nicole J. van Steijn … Michiel M. Winter

**Supplementary material**

**Address for correspondence:** Nicole J. van Steijn, Amsterdam UMC location AMC, 1105 AZ Amsterdam, The Netherlands; +31616680899, [njvansteijn@gmail.com](mailto:njvansteijn@gmail.com)

**Contents**

**Supplementary Tables**………………………………………………………………………………..3

Table S1………………………………………………………………………………………………...3

Table S2………………………………………………………………………………………………...4

**Supplementary Figures**……………………………………………………………………………….5

Figure S1………………………………………………………………………………………………..5

**Supplementary Tables**

**Supplementary Table S1. Reasons for exclusion of 12-lead and mobile ECGs**

This table lists the reasons for exclusion of 12-lead and mobile ECGs from analysis. ECGs were excluded due to flat T waves or signal artefacts.

|  |  | Recorded | | Flat T waves | Artefacts | |  |
| --- | --- | --- | --- | --- | --- | --- | --- |
| Lead I | 12-lead  mECG | 101 101 | 0 (0) 2 (2.0) | | 0 (0)  1 (1.0) |  | |
| Lead II | 12-lead  mECG | 101 101 | 2 (2.0) 4 (4.0) | | 1 (1.0) 2 (2.0) | |  |
| Values are presented as numbers and percentages. mECG, mobile electrocardiogram. | | | | | | | |

**Supplementary Table S2. Agreement Between 12-lead and mobile ECG–Derived QTc values per genotype**

This table presents the agreement in QTc measurements between the 12-lead ECG and mobile ECG device, stratified by cLQTS genotype. Results are shown separately for recordings obtained in lead I and lead II positions, and include mean differences and limits of agreement for each subgroup.

|  | Patients (n) | Lead I  (mean difference; 95% LOA) | Patients (n) | | Lead II  (mean difference; 95% LOA) |
| --- | --- | --- | --- | --- | --- |
| *KCNQ1* | 34 | -21.8 (-52.2 to 8.6) | 34 | -23.3 (-60.8 to 14.1) | |
| *KCNH2* | 40 | -22.0 (-52.3 to 8.3) | 37 | -21.6 (-54.2 to 10.9) | |
| *SCN5A* | 20 | -20.4 (-59.2 to 18.4) | 18 | -16.6 (-67.5 to 34.2) | |
| *CACNA1C* | 3 | -22.5 (-35.3 to – 9.74) | 3 | -18 (-68.3 to 32.3) | |
| *KCNE1* | 1 | -26.8 | 1 | -4.7 | |
| Mean differences were calculated as the QTc measurement from 12-lead ECG minus the corresponding mobile ECG value.  LOA were calculated where applicable. For the KCNE1 gene, as only one patient was available, differences are presented without LOA.  LOA, Limits of agreement. | | | | | |

**Supplementary Figures**

**
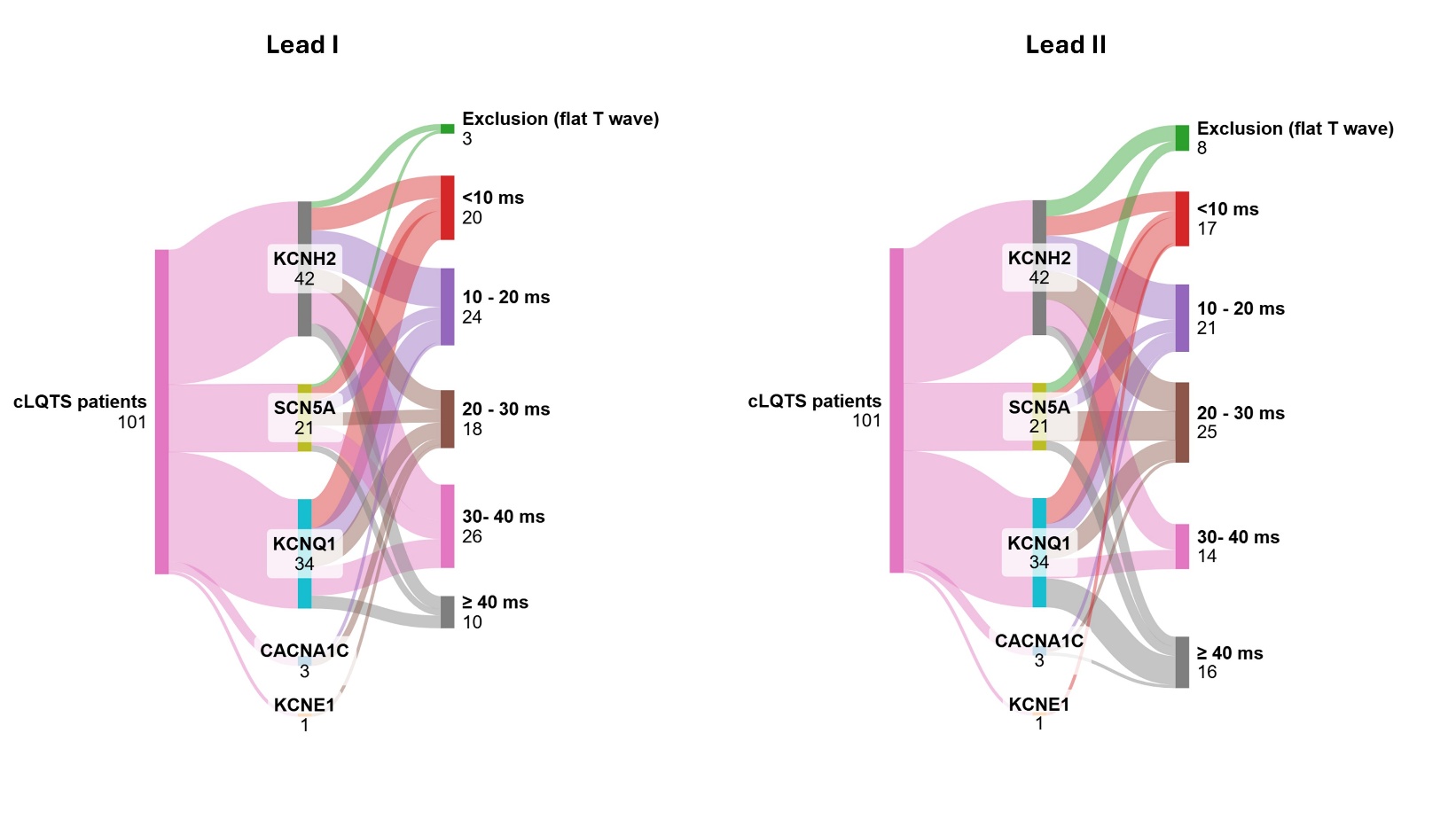
Figure S1.** **Flow of QTc measurements across agreement categories**

**Figure S1.** Sankey diagrams illustrating the distribution of QTc agreement categories between 12-lead ECG and Apple Watch mECG for leads I (left) and II (right).

On the left side of each diagram, the total number of patients is shown, which divides into the five genotype groups (*KCNH2*, *KCNQ1*, *SCN5A*, *CACNA1C*, and *KCNE1*). The right side displays the number of patients within each QTc agreement category (<10 ms, 10–20 ms,
20–30 ms, 30–40 ms, ≥40 ms). Flow width represents the number of patients per connection. Mean difference is defined as 12-lead QTc minus Apple Watch QTc.

Abbreviations: cLQTS, congenital Long QT syndrome; ms, milliseconds.
